# Predictors of recurrence after pulmonary vein isolation in patients with normal left atrial diameter

**DOI:** 10.1002/joa3.12230

**Published:** 2019-09-02

**Authors:** Masamichi Yano, Yasuyuki Egami, Kyosuke Yanagawa, Yutaka Matsuhiro, Hitoshi Nakamura, Koji Yasumoto, Naotaka Okamoto, Akihiro Tanaka, Yasuharu Matsunaga‐Lee, Daisuke Nakamura, Masaki Yamato, Ryu Shutta, Masami Nishino, Jun Tanouchi

**Affiliations:** ^1^ Division of Cardiology Osaka Rosai Hospital Osaka Japan

**Keywords:** atrial fibrillation, normal left atrial diameter, pulmonary vein isolation, recurrence

## Abstract

**Background:**

Enlarged left atrium (LA) is an established predictor of recurrence of atrial fibrillation (AF) after pulmonary vein isolation (PVI), but occasionally recurrences of AF/atrial tachycardia (AT) are experienced in patients with normal left atrial diameter. Therefore, the predictors of AF recurrence and AF triggers were evaluated in patients with normal LA.

**Methods:**

We enrolled 168 patients with normal LA (<40 mm) who underwent PVI. Various predictors were compared, including age, gender, coronary risk factors, brain natriuretic peptide (BNP), medications, echocardiographic parameters, and procedure parameters, between recurrence and nonrecurrence groups.

**Results:**

The recurrence group consisted of 50 patients (29.8%). A univariate analysis demonstrated that the ratio of females, high BNP levels, severe tricuspid valve regurgitation (TR), and relapses of AF/AT during catheter ablation (CA) were significantly higher in the recurrence group. Multivariate analyses showed that a high BNP, severe TR, and AF/AT relapses during CA were independent factors associated with AF recurrence. During the second CA sessions, nonpulmonary vein (PV) triggers were therapeutic targets in 18 patients (46.2%), which was higher than that previously reported.

**Conclusion:**

A high BNP, severe TR and AF/AT relapses during CA may be correlated with AF recurrence after PVI in the patients with normal LA.

## Graphical abstract

We investigated the predictors of atrial fibrillation (AF) recurrence in patients with normal left atrium (LA). A high brain natriuretic peptide, severe tricuspid valve regurgitation and AF/atrial tachycardia relapses during ablation may be correlated with AF recurrence after ablation in the patients with normal LA.

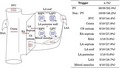

## 1 INTRODUCTION

Atrial fibrillation (AF) is the most sustained arrhythmia. Since extrasystoles from pulmonary veins (PV) are the most common AF trigger activity.^1^ pulmonary vein isolation (PVI) has become established as the main therapy for patients with drug‐refractory paroxysmal atrial fibrillation (PAF).^2^, ^3^, ^4^, ^5^ The efficacy and safety of PVI for AF has been reported in several randomized trials.^6^, ^7^, ^8^, ^9^ There are many studies that have evaluated the predictors of recurrences after PVI.^10^, ^11^, ^12^, ^13^ Left atrial (LA) enlargement is an independent risk factor for a recurrence after PVI.^11^, ^14^, ^15^ However, we occasionally experienced AF/atrial tachycardia (AT) recurrences after PVI in patients with non‐enlarged LA, that is to say, normal left atrial diameter. The characteristics of AF recurrence in patients with a normal LA remain unclear. In this study, we evaluated the predictors of AF recurrence and AF triggers in patients with normal LAs.

## 2 METHODS

### 2.1 Study populations

We enrolled consecutive patients with a normal LA (<40 mm) who underwent PVI between April 2010 and December 2015. All patients received a detailed informed consent and the study protocol was approved by the hospital's institutional review board.

### 2.2 Echocardiography study

All patients underwent transthoracic echocardiography before the catheter ablation (CA). Transthoracic echocardiography was performed with a 5 MHz multiplane probe and live images were interpreted by experienced physicians who were blinded to the outcome of the CA. The left atrial diameter was measured at end‐systole on the M‐mode image obtained from the parasternal long‐axis view. Mitral regurgitation and tricuspid regurgitation (TR) were graded as none/trace, mild, moderate, or severe. Transesophageal echocardiography prior to the AF ablation was performed to exclude any LA and LA appendage thrombi.

### 2.3 Ablation procedure

All antiarrhythmic drugs were discontinued for at least five half‐lives before the ablation. Anticoagulation therapy was started at least 3 weeks before the ablation procedure. A bolus infusion of hydroxyzine pamoate 25 mg and pentazocine 15 mg was intravenously administered before the ablation procedure. The ablation procedure was performed under mild sedation obtained with propofol and dexmedetomidine and the patients received adaptive servoventilation (ASV). An esophageal temperature monitoring catheter was placed via the nose. A duo‐decapolar catheter (BeeAT, Japan Lifeline Co.) was placed in the coronary sinus (CS) through the right internal jugular vein. If the patient's rhythm was AF, internal atrial cardioversion was performed with a biphasic energy of 15‐20 J. The transseptal puncture was performed under guidance with a SoundStar 3D Ultrasound Catheter (Biosense Webster) from the right atrium (RA). After the transseptal puncture, 2 long sheaths (SL0; St Jude Medical) were inserted into the LA. A 100 IU/kg body weight of heparin was administered following the transseptal puncture and heparinized saline was continuously infused to maintain the activated clotting time at 300‐350 seconds. One or two circular mapping catheters were deployed in the superior and inferior PVs and the left‐sided then the right‐sided ipsilateral PVs were circumferentially ablated guided by three‐dimensional (3D) LA mapping (CARTO3, Biosense‐Webster). The PVI was performed with no contact force (CF) catheter (THERMOCOOL SF ^®^ Catheter; Biosense‐Webster) (no contact force (CF) catheter) or CF catheter (THERMOCOOL SMARTTOUCH^®^ Catheter; Biosense‐Webster). Radiofrequency current was delivered with power up to 30 W and limited to 20 W near the esophagus for 25 seconds. The endpoint of PVI was the achievement of bidirectional conduction block between the LA and PVs, and any dormant PV conduction revealed by adenosine triphosphate (ATP) and isoproterenol was eliminated. PVI was considered successful when all ostial PV potentials were abolished during coronary sinus pacing and was also confirmed by PV pacing under isoproterenol (until increasing heart rate) and ATP bolus administration (40 mg). Cavo‐tricuspid isthmus block was created in almost all patients. When AF persisted after the PVI or firing sites of APC triggers were detected, substrate modification was sequentially performed. A second ablation session was performed when a relapse of AF/AT occurred 3 months after the PVI and PV reconnections and non‐PV foci were confirmed. In repeated ablation, to detect non‐PV foci, we attempted to locate the spontaneous onset of ectopic beats initiating AF in the baseline state or after infusion of isoproterenol (up to 4 μg/min).

### 2.4 Follow‐up

After the CA, no antiarrhythmic medications were prescribed. The patients underwent continuous electrocardiogram (ECG) monitoring for approximately 3 days (until discharge). BNP and creatinine were measured at 2 days after PVI. They visited our cardiology clinic 1 month after the ablation. Subsequent follow‐up visits were performed every 3 months at the clinic. The follow‐up visit included a clinical interview, ECG, blood examination, 24 hour Holter monitoring or a portable ECG (2‐week cardiac event recording), and echocardiography. Patients with palpitations or other chest symptoms underwent a portable ECG. Recurrence after the CA was defined as AF/AT documented on the ECG or AF/AT continuing longer than 30 seconds on the Holter or portable ECG. AF/AT for first 3 months after the PVI (blanking period) was not considered as a recurrence.

### 2.5 Statistical analysis

JMP 11 statistical software was used for the statistical analysis. The continuous parameters are expressed as mean ± SD. Two‐group comparisons were analyzed using an unpaired 2‐tailed Student's *t* test. Categorical data were expressed as the number (percentage) and were compared using a Chi‐square test. Parameters with a significance of <0.05 in the univariate analysis were entered into Cox proportional hazard analysis.

## 3 RESULTS

### 3.1 Patient characteristics and outcomes after ablation

In 549 consecutive patients with PVI, the patients with a normal LA (<40 mm) consisted of 168 patients (30.6%) during the study period. During the follow‐up period, 50 of 168 patients suffered from AF/AT recurrence after ablation. The clinical characteristics of the patients in the recurrence and no recurrence groups are shown in Table 1. The ratio of females and the serum BNP level were higher in the recurrence group than no recurrence group (*P* = .030 and .041, respectively). Regarding the echocardiographic data, the ratio of severe TR was higher in the recurrence group than no recurrence group (*P* = .014). Regarding TR, eight cases (three moderate TR cases and five severe TR cases) underwent 2nd session CA. In severe TR cases, four of five (80%) right‐sided non‐PV triggers were confirmed and seven of eight (87.5%) TR patients (moderate and severe TR) were confirmed as right‐sided non‐PV triggers (Table 2). None of the other parameters differed between the two groups.

**Table 1:** Patient characteristics

	Recurrence (‐) N = 118	Recurrence (+) N = 50	*P*	
Age, y	63.4 ± 10.1	63.5 ± 9.54	.925	
BMI, kg/m^2^	22.2 ± 3.64	22.1 ± 3.09	.889	
Female, n (%)	38 (32.2%)	25 (50.0%)	.030	
CHF, n (%)	9 (7.7%)	3 (6.0%)	.708	
Hypertension, n (%)	44 (37.2%)	16 (32.0%)	.513	
Diabetes mellitus, n (%)	11 (9.3%)	2 (4.0%)	.238	
Stroke, n (%)	4 (3.4%)	5 (10.0%)	.085	
Hyperthyroidism, n (%)	0 (0%)	0 (0%)	—	
Smoking, n (%)	23 (19.5%)	11(22.0%)	.711	
CAD, n (%)	5 (5.9%)	5 (10.0%)	.335	
PAF, n (%)	110 (93.2%)	44 (88.0%)	.263	
CHADS2 score				
0, 1, n (%)	102 (86.4%)	43 (86.0%)		.110
2, n (%)	12 (10.2%)	2 (4.0%)		
>3, n (%)	4 (3.4%)	5 (10.0%)		
CHADS2‐Vasc score				
0, 1, n (%)	89 (75.4%)	33 (66.0%)		.333
2, n (%)	17 (14.4%)	12 (24.0%)		
>3, n (%)	12 (10.2%)	5 (10.0%)		
BNP (pg/mL)	61.3 ± 74.4	101.7 ± 173.9		.041
Creatinine	0.9 ± 0.8	0.9 ± 1.0		.910
Echocardiogram				
LVDd, mm	46.7 ± 3.90	45.7 ± 3.90		.108
LVDs, mm	28.1 ± 3.85	27.9 ± 3.81		.752
LVEF, %	69.8 ± 7.13	68.9 ± 8.81		.507
LADs, mm	36.6 ± 3.41	37.0 ± 2.42		.484
Severe MR, n (%)	1 (0.9%)	0 (0%)		.514
Severe TR, n (%)	2 (1.7%)	5 (10.0%)		.014
TRPG, mm Hg	24.0 ± 4.75	26.8 ± 5.95		.282
Mean TDI E/e’	9.50 ± 2.88	11.0 ± 5.83		.039

Abbreviations: BNP: brain natriuretic peptide; CAD, coronary artery disease; CHF, chronic heart failure; LAD: left atrial dimension; LVDd: left ventricular dimension at diastole; LVD: left ventricular dimension at systole; LVEF: left ventricular ejection fraction; MR: mitral valve regurgitation; PAF: paroxysmal atrial fibrillation; TR: tricuspid valve regurgitation.

**Table 2:** Right‐sided non‐PV trigger location in TR patients

Case	Non‐PV triggers
Moderate TR	
Case1	SVC
Case2	SVC
Case3	RA (high lateral)
Severe TR	
Case1	SVC, RA (high lateral)
Case2	SVC
Case3	None
Case4	RA septum, RAA
Case5	RA septum

Abbreviations: RA, right atrium; RAA, right atrial appendage; SVC, superior vena cava; TR, tricuspid regurgitation.

### 3.2 Oral medications

The oral medications before the CA are shown in Table 3. The ratio of angiotensin‐converting‐enzyme inhibitors, angiotensin II receptor blockers, diuretics, statins, oral anticoagulants (warfarin and direct oral anticoagulant), and antiarrhythmic drugs did not differ significantly between the two groups.

**Table 3:** Oral medications

	Recurrence (−) N = 118	Recurrence (+) N = 50	*P*
ACE Inhibitor, n (%)	8 (6.8%)	1 (2.0%)	.208
ARB, n (%)	16 (13.6%)	3 (6.0%)	.157
Diuretics, n (%)	8 (6.8%)	3 (6.0%)	.852
Digitalis, n (%)	2 (1.7%)	2 (4.0%)	.37
Statin, n (%)	20 (17.0%)	11 (22.0%)	.44
Oral anticoagulant, n (%)			
Warfarin	55 (46.6%)	27 (54.0%)	.381
Direct oral anticoagulant	63 (53.4%)	22 (44.0%)	.266
Anti‐arrhythmic drug, n (%)			
Class Ia	17 (14.4%)	10 (20.0%)	.367
Class Ic	20 (17.0%)	6 (12.0%)	.417
Class II (βblocker)	36 (30.5%)	18 (36.0%)	.486
Class IV (Ca antagonist)	15 (12.7%)	7 (14.0%)	.821
Class IV (Bepridil)	10 (8.47%)	7 (14.0%)	.278

Abbreviations: ACE, angiotensin‐converting‐enzyme; ARB, angiotensin II receptor blocker.

### 3.3 Procedure characteristics

The procedural characteristics of the patients in the recurrence and no recurrence groups are shown in Table 4. Episodes of AF/AT during the CA, including spontaneous AF/AT episodes of any duration observed during the first‐time ablation, were greater (*P* < .001) and the ratio of the intracardiac direct current had a tendency to be higher in the recurrence group than no recurrence group (*P* = .078) There was no significant difference in any of the other parameters between the two groups. We performed PVI using no CF and CF catheter because of the difference of CA period. In all 168 patients, 64 patietns underwent PVI using no CF catheter. The incidence of recurrence using no CF catheter was 29.7% (19/64) and that using CF catheter was 29.8 % (31/104) (no significant difference).

**Table 4:** Procedural characteristics

	Recurrence (−) N = 118	Recurrence (+) N = 50	*P*
Cavo tricuspid isthmus block, n (%)	86 (72.9%)	33 (66.0%)	.37
Ablation of left atrium substrate, n (%)	25 (21.2%)	13 (26.0%)	.495
Intracardiac direct current cardioversion, n (%)	21 (17.8%)	15 (30.0%)	.078
Episodes of AF/AT during CA, n (%)	42 (35.6%)	32 (64.0%)	<.001
Total number of energy applications	73.2 ± 35.8	75.7 ± 41.2	.747

Abbreviations: AF, atrial fibrillation; AT, atrial tachycardia; CA, catheter ablation.

### 3.4 Predictors of AF/AT recurrence after PVI

**Figure 1:**
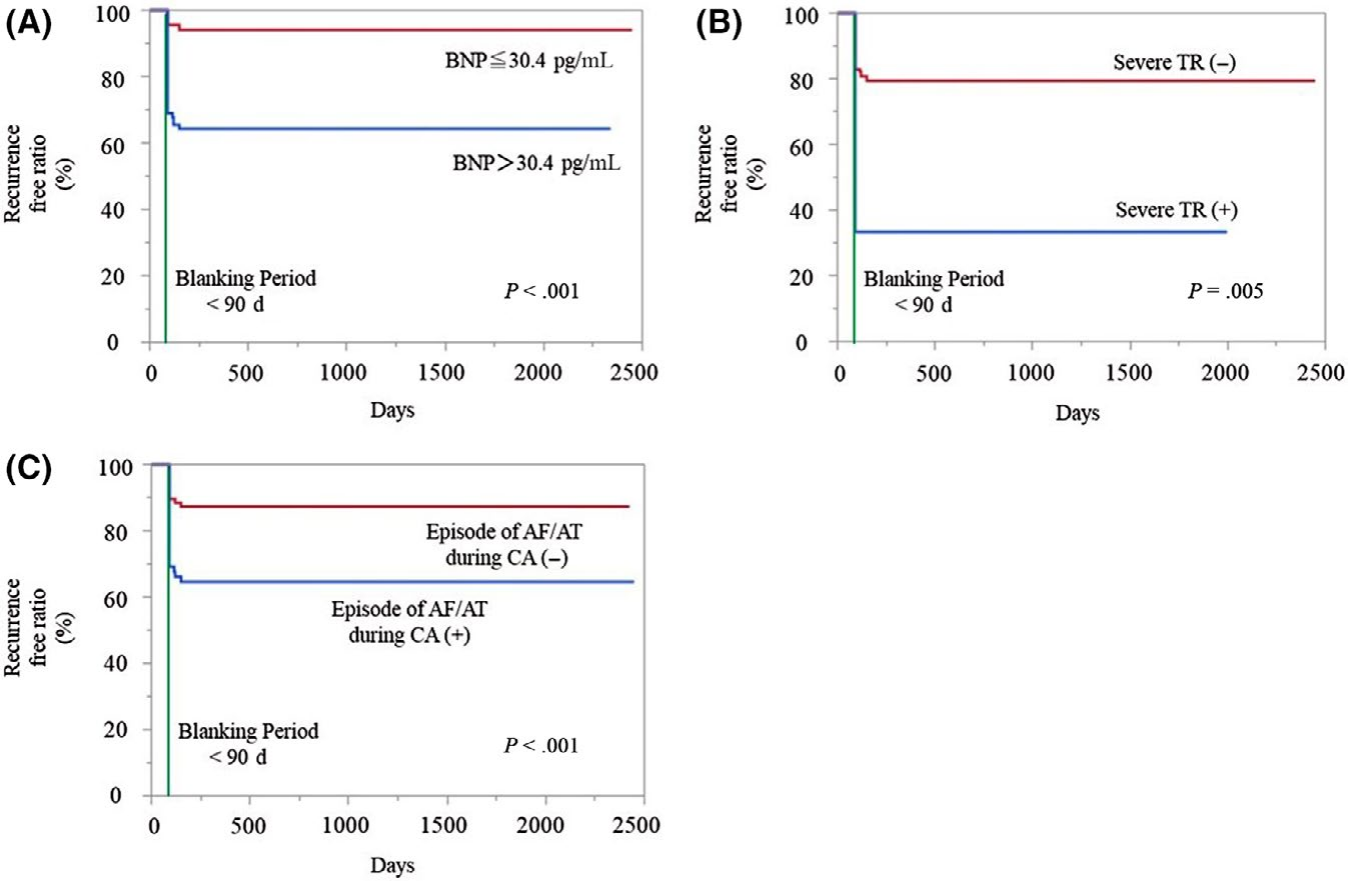
Difference in the incidence of AF recurrence between BNP >30.4 pg/mL vs BNP of ≤30.4 pg/mL (A), between the presence vs. absence of severe TR (B), and between the presence vs. absence of episode of AF/AT during CA (C) (Kaplan‐Meier curve). AF, atrial fibrillation; AT, atrial tachycardia; BNP, brain natriuretic peptide

A receiver operating characteristics analysis revealed a good accuracy of predicting a recurrence by the BNP (AUC‐ROC: 0.633). With a cut‐off of 30.4 pg/mL for the BNP, a sensitivity of 77% and specificity of 52% were achieved. A univariate analysis demonstrated that the ratio of females, a high BNP level (>30.4 pg/mL), severe TR, and relapses of AF/AT during the CA were significantly higher in the recurrence group (*P* = .029, .006, .014 and .001 respectively), however, there was no significant difference in the other predictors between the two groups. A multivariate analysis showed that a high BNP, severe TR, and relapses of AF/AT during the CA were independent factors associated with AF recurrence (*P* = .002, .047, and .001. respectively) (Table 5). Kaplan‐Meier curve for AF recurrence after PVI between BNP >30.4 pg/mL vs BNP of ≤30.4 pg/mL, between the presence vs. absence of severe TR, and between the presence vs absence of episode of AF/AT during CA (analysis of those comparisons by Log‐rank test) were shown in Figure 1 (*P* < .001, *P* < .001, *P* = .005, respectively).

**Table 5:** Factors associated with atrial fibrillation recurrence

	OR	95% CI	*P*
BNP (>30.4 pg/mL)	2.761	1.415‐5.811	.002	
Severe TR	3.008	1.016‐7.175	.047	
Episode of AF/AT during CA	2.632	1.470‐4.878	.001	

Abbreviations: CI, confidence interval; OR, odd's ratio. The other abbreviations are same as Tables 1 and 3.

### 3.5 Effect of early recurrence and antiarrhythmic drugs on late recurrences after PVI

AF/AT recurrences during the first 3 months after the PVI (blanking period) occurred in 46 patients (27.3%). Thirty‐two of 46 patients (69.6%) had late recurrences after the PVI, and 18 of 122 (14.8%), who presented with no recurrences for 3 months after the PVI, had late recurrences after the PVI (69.6% vs 14.8%, *P* < .001). Twenty‐eight of 46 patients were treated with antiarrhythmic drugs and 18 without them. Nineteen of 28 patients with antiarrhythmic drugs and 13 of 18 without antiarrhythmic drugs had late recurrences, respectively (60.9% vs 72.2%, *P* = .754).

### 3.6 Therapeutic targets of AF/AT triggers in second ablation session

**Figure 2:**
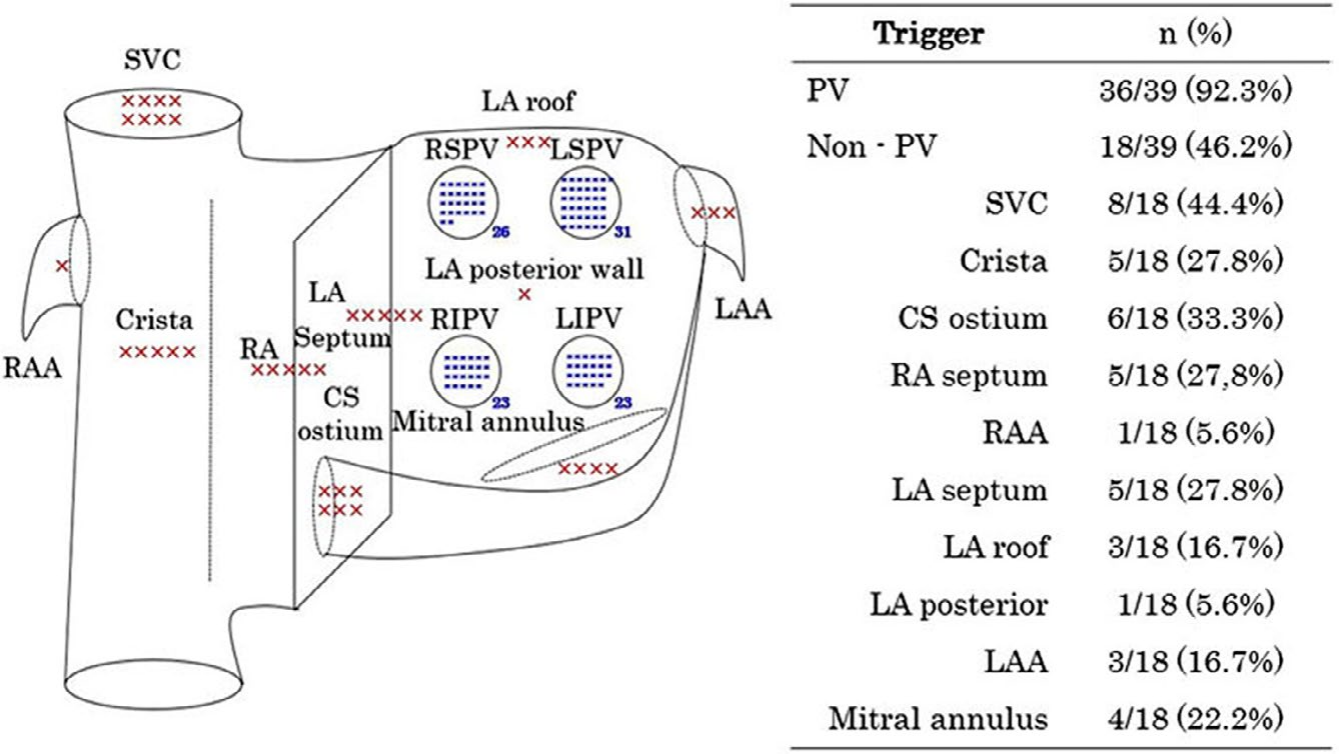
Therapeutic targets of AF/AT triggers in repeated ablation procedures. AF, atrial fibrillation; AT, atrial tachycardia

Repeated ablation procedures were performed in 39 of 50 patients who had AF/AT recurrences after the PVI. Pulmonary vein triggers were observed in 36 patients (92.3%) and nonpulmonary vein triggers were therapeutic targets in 18 (46.2%), which was higher than that previously reported.^16^ Many non‐PV foci originated from the superior vena cava, crista terminalis, ostium of the coronary sinus, and the right atrial septum (Figure 2).

## 4 DISCUSSION

There are many studies that have evaluated the predictors of recurrences after PVI. Those reports have referred to LA overload‐associated factors, such as LA size, LA pressure, and LA volume (index), as independent predictors of AF after PVI.^17^, ^18^, ^19^ In this study, we focused on AF patients with a normal LA who were assumed not to have had a relapse of AF after the PVI. Difference of catheter could not affect the ratio of AF/AT recurrence after PVI. To the best of our knowledge, this is the first study to demonstrate that the independent predictive factors of AF recurrences after PVI were a high serum BNP level, severe TR, and relapses of AF/AT during the CA in patients with a normal LA.

### 4.1 BNP level

In general, patients with a normal LA have a normal atrial compliance and contractility. However, a normal LA with AF might reduce the atrial systolic or diastolic function and cannot compensate for an elevated pulmonary capillary wedge pressure because of an LA substrate abnormality. Therefore, the BNP secretion may increase in the recurrent group.^20^ PV reconnection rate after PVI in our study was 92.3%. Previous reports demonstrated that the PV reconnection rate after first PVI was 86% in patients with PAF including various LA dimensions.^21^ PV reconnection is the most important factor for initiating and maintaining AF due to structural and electrical remodeling of LA. In this study we showed high BNP level, which reflected promoting LA remodeling, was one of the risk factor for AF recurrence and might cause PV reconnection. Previous reports demonstrated biomarkers such as BNP (NT‐proBNP) that were involved in the development and recurrence of AF.^22^, ^23^ A recent study showed that PV reconnection rate after first PVI was 86% in patients with PAF including various LA dimensions.^21^ Generally left atrium‐pulmonary vein reconnection after PVI is caused by insufficient transmural ablation because of anatomical difficulty^24^ and edematous tissue after ablation.^25^ High BNP, suggesting left atrial overload, promotes activation of sympathetic nerve and enhances automaticity of ectopic excitation in PV.^26^ These ectopic excitations may increase conduction through PV‐LA. Left atrial overload also promotes proinflammatory and profibrotic pathways and promotes structural and electrical remodeling, as a result, AF is maintained in LA.

### 4.2 Severe TR

Severe TR was an independent predictive factor of AF/AT recurrence after PVI in this study. In the second ablation session, PV reconnections were observed in most patients (92.3%) and also non‐PV foci, especially in right‐sided areas (superior vena cava, crista terminalis, ostium of coronary sinus and right atrial septum), were observed in many patients (Figure 1). These results suggest the relationship between TR and right‐sided non‐PV trigger. A previous report demonstrated that the voltage in the RA was low with RA atypical flutter and RA‐AF.^27^ In the present study, severe TR promoted RA substrate abnormalities and right‐sided heart dysfunction might be related to the substrate changes and subsequent right‐sided non‐PV triggers.

### 4.3 Relapses of AF/AT during CA

We showed that relapses of AF/AT during CA were one of the independent factors of AF recurrence in the patients with a normal LA. In 168 patients with a normal LA, relapses of AF/AT were detected in 74 patients and 29 (39.2%) underwent non‐PV foci ablation, whereas in 94 patients, no relapses of AF/AT were detected and only nine patients (9.6%) underwent a non‐PV foci ablation (*P* < .001). LA substrate abnormalities promote automaticity of atrial cells and atrial tissue fibrosis, conduction slowing, and refractory period changes that occur with LA substrate instability that forms micro‐reentries and maintains fibrillation.^28^ The relapses suggested that there were atrial substrate abnormalities and predicted non‐PV foci as triggers of AF.

## 5 CONCLUSIONS

A high BNP level, severe TR, and relapses of AF/AT during CA may be correlated with the recurrence of AF after the PVI in the patients with a normal LA. In addition, the incidence of non‐PV foci as a trigger during a second CA session was higher in those patients.

## CONFLICT OF INTEREST

Authors declare no conflict of interests for this article.
